# Immediate postnatal care following childbirth in Ugandan health facilities: an analysis of Demographic and Health Surveys between 2001 and 2016

**DOI:** 10.1136/bmjgh-2020-004230

**Published:** 2021-04-22

**Authors:** Teesta Dey, Sam Ononge, Andrew Weeks, Lenka Benova

**Affiliations:** 1 Women's and Children's Health, University of Liverpool, Liverpool, UK; 2 Department of Obstetrics and Gynaecology, Makerere University, Kampala, Uganda; 3 Department of Public Health, Institute of Tropical Medicine, Antwerpen, Belgium

**Keywords:** maternal health, obstetrics

## Abstract

**Introduction:**

Progress in reducing maternal and neonatal mortality, particularly in sub-Saharan Africa, is insufficient to achieve the Sustainable Developmental Goals by 2030. The first 24 hours following childbirth (immediate postnatal period), where the majority of morbidity and mortality occurs, is critical for mothers and babies. In Uganda,<50% of women reported receiving such care. This paper describes the coverage, changes over time and determinants of immediate postnatal care in Uganda after facility births between 2001 and 2016.

**Methods:**

We analysed the 2006, 2011 and 2016 Ugandan Demographic and Health Surveys, including women 15–49 years with most recent live birth in a healthcare facility during the survey 5-year recall period. Immediate postnatal care coverage and changes over time were presented descriptively. Multivariable logistic regression was used to examine determinants of immediate postnatal care.

**Results:**

Data from 12 872 mothers were analysed. Between 2006 and 2016, births in healthcare facilities increased from 44.6% (95% CI: 41.9% to 47.3%) to 75.2% (95% CI: 73.4% to 77.0%) and coverage of immediate maternal postnatal care from 35.7% (95% CI 33.4% to 38.1%) to 65.0% (95% CI: 63.2% to 66.7%). The majority of first checks occurred between 1 and 4 hours post partum; the median time reduced from 4 hours to 1 hour. The most important factor associated with receipt of immediate postnatal care was women having a caesarean section birth adjusted OR (aOR) 2.93 (95% CI: 2.28 to 3.75). Other significant factors included exposure to mass media aOR 1.38 (95% CI: 1.15 to 1.65), baby being weighed at birth aOR 1.84 (95% CI: 1.58 to 2.14) and receipt of antenatal care with 4+Antenatal visits aOR 2.34 (95% CI: 1.50 to 3.64).

**Conclusion:**

In Uganda, a large gap in coverage remains and universal immediate postnatal care has not materialised through increasing facility-based births or longer length of stay. To ensure universal coverage of high-quality care during this critical time, we recommend that maternal and newborn services should be integrated and actively involve mothers and their partners.

**Key questions**

**What is already known?**

- Postnatal care has the poorest coverage levels in the obstetric continuum of care.
- The highest maternal and newborn morbidity and mortality occurs in the immediate postnatal period (within 24 hours of birth)

**What are the new findings?**

- Although there are increases in the proportion of births occurring in health facilities, and immediate postnatal care coverage after facility birth has doubled, coverage remains suboptimal over the time period examined.
- In facility births, the timing of the first postnatal check has shifted closer to the time of birth.
- Women/newborns with complications and those with higher social status are more likely to report receiving immediate postnatal checks.

**What do the new findings imply?**

- There is need to improve the coverage of immediate postnatal care checks
- It is important to establish guidance on the optimal timing for postnatal maternal checks.
- New strategies are needed to increase coverage of high-quality postnatal care, for example, integration of maternal and newborn care services, or active involvement of mothers and birth partners.

## Introduction

Globally, since 2000, maternal and neonatal mortality rates have declined significantly and rates are currently 211 per 100 000 live births and 18 per 1000 live births, respectively.^1 2^ Although encouraging, this progress is insufficient to achieve the Sustainable Development Goals of 70 maternal deaths per 100 000 live births and 12 neonatal deaths per 1000 live births by 2030. Moreover, the majority of countries failing to reach these levels are in sub-Saharan Africa.^3 4^ Over the last 10 years, work has been done to improve the continuum of care provided to mothers before and during childbirth through increasing regular, skilled antenatal clinic provision and facility-based deliveries. This may have contributed to the steady decline of maternal and neonatal mortality rates.^5 6^ However, postnatal care is often neglected and overlooked.^7 8^

Most maternal and newborn mortality occurs between the time of birth and the first 2 days post partum, and a focus on the immediate postnatal period is therefore important.^9 10^ Key causes of maternal mortality include postpartum haemorrhage, hypertensive disorders, pre-eclampsia and sepsis.^11^ Key causes of newborn mortality include preterm birth complications, infection and intrapartum conditions such as asphyxia.^12^ The vast majority of cases of maternal and newborn mortality are treatable and preventable but require quick recognition and good-quality care.^13 14^ As such, there has been a push to increase the number of women delivering in healthcare facilities in low-resource settings in the hope that this will help increase coverage of care during birth and the ongoing postpartum period.^14^ Postnatal care is care provided to both mother and baby following childbirth, and is recommended to continue regularly for 6 weeks.^15^ Immediate postnatal care is the initial care provided by trained health workers to women in the first 24 hours following birth of baby and can be provided at home or within a health facility depending on place of birth. It is a critical time period in childbirth where maternal and newborn mortality and morbidity is the highest, particularly in sub-Saharan Africa.^9 11^

Postnatal care for a newborn baby involves ensuring good feeding, assessing vital signs, examining the umbilical stump and checking for sepsis and jaundice. For mothers, these checks involve measuring vital signs, assessing and addressing any physical symptoms that the mother is experiencing which may indicate severe conditions, assessing uterine contraction, vaginal tears/discharge or caesarean incision sites, assessing their ability to urinate and defecate, screening for postnatal depression and conducting any other assessments based on existing conditions. Conducting this care is pertinent as it not only allows these assessments to occur and any treatment to be initiated, if needed, but provides an opportunity for health workers to provide advice and counselling on breastfeeding and newborn care. For facility-based births, WHO has advised that for at least 24 hours following vaginal delivery, women should be observed and cared for in the facility where they gave birth, to enable mothers and babies to receive this vital care.^15^ For home births, the first postnatal check should occur within 24 hours of birth. Thereafter, all mothers and newborns should receive at least three additional postnatal checks on day 3, between day 7 and 14, and between week 2 and week 6 following birth of baby.^15^

Despite the increase in healthcare facility births, in many low resource settings>75% of mothers do not receive the adequate postnatal health checks they require^16 17^ In Uganda, over the past 30 years, there has been a considerable shift in the health system arrangements. In the early 1990s, Uganda implemented a widescale public sector reform in line with the World Banks Structural Adjustment programme.^18^ This resulted in a widescale downsizing and decentralising of health services increasing access. Public health services are free allowing more women to access care.^19^ Additionally, strong political will, donor funding and a rise in non-profit facilities have been highlighted as key contributors to resource mobilisation maternal health prioritisation.^20 21^ As such, promising progress has been made to improve the percentage of births occurring in health facilities, from 37% in 2000 to 73% in 2016.^5^ This has coincided with a 30% decline in maternal mortality rates since 1988.^5^ However, despite these efforts, the maternal mortality rate in 2017 remained high at 336/100 000 live births and adequate postnatal care remains the poorest performing aspect of the obstetric continuum in Uganda.^5^ National guidance from the Ugandan Ministry of Health for postnatal care exists within the Uganda Clinical Guideline which were created adopting WHO recommendations.^22^ However, most recent estimates indicate that the provision of this vital care occurred in less than 50% of facility births making it the fifth worst performing country out of the 33 sub-Saharan African countries examined.^17^ It is clear that change is needed to enable equity in coverage of postnatal care.

Existing global research has often focused on postnatal care coverage among rural and home-based births or all births and there is little literature specific to care following facility births in Uganda.^23–25^ Additionally, the intersection between maternal and newborn postnatal care coverage has not been described in previous work. Given the lack of improvement to postnatal care coverage from healthcare facility births despite the large shift toward facility-based childbirth, we sought to understand the coverage, changes over time and determinants of immediate postnatal care in Uganda after facility births over the past 15 years.

In this manuscript, we look to describe the coverage and timing of immediate postnatal care for mothers following childbirth in healthcare facilities in Uganda using three Ugandan Demographic Health Surveys (2006, 2011, 2016). Additionally, we analysed the determinants of immediate maternal postnatal care following deliveries in healthcare facilities on the 2016 survey.

## Methods

### Data

Household surveys are the main source of data used within maternal health to compare coverage trends and inequalities both within and between countries.^26^ The Demographic and Health Surveys (DHS) are cross-sectional nationally representative household surveys, usually covering 5000–30 000 households. They collect data from women in reproductive age (15–49 years) about births and the use of reproductive and maternal care. We used the DHS collected in Uganda in 2006, 2011 and 2016. The DHS use a multilevel cluster sampling survey design; individual women’s survey weights, and the elements of stratification and clustering are needed in analysis to adjust for this design and for non-response.

### Population

**Figure 1:**
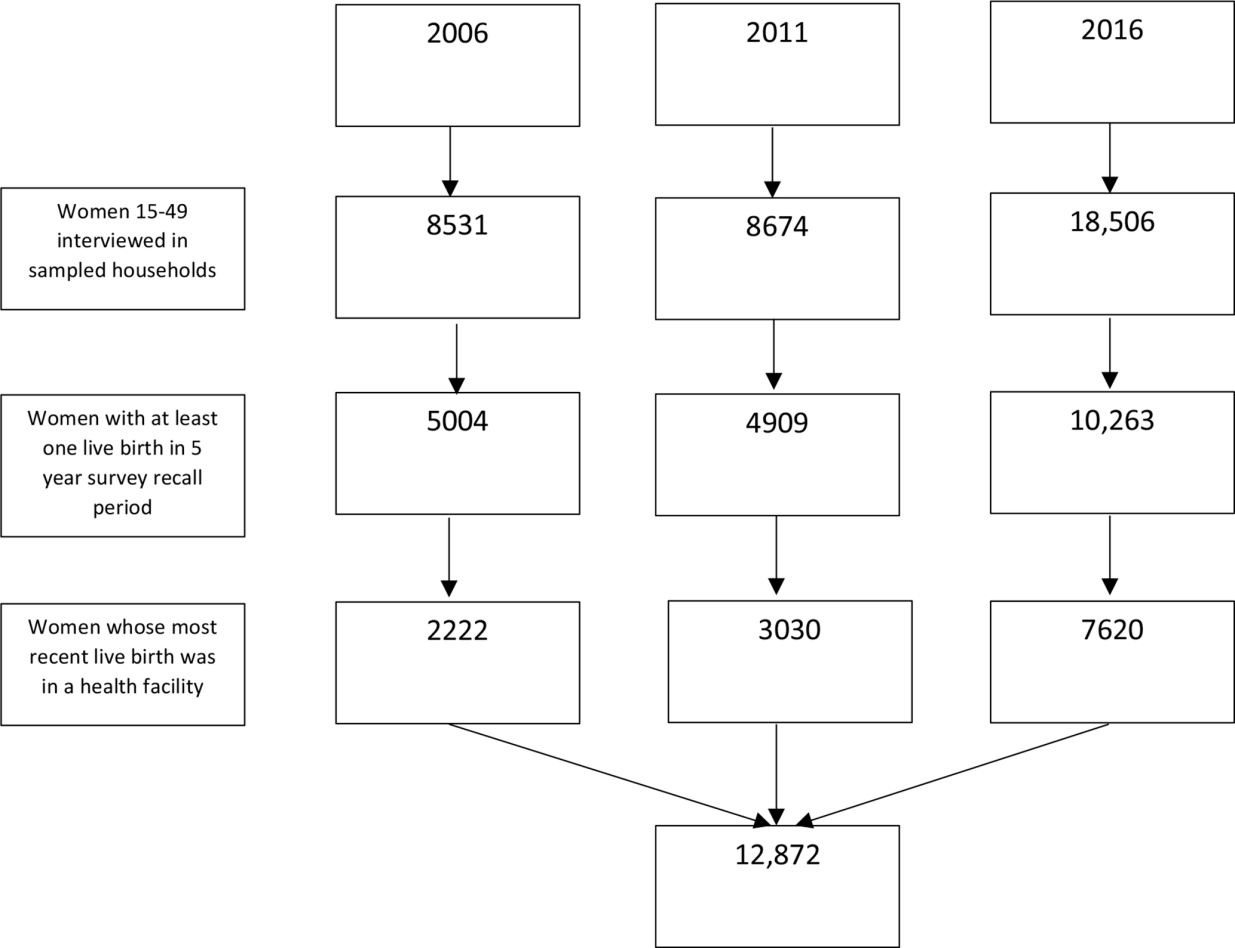
Study population flow diagram.

The most recent live birth within a recall period of 5 years to women aged 15–49 at the time of survey, was included in the analysis, if the birth occurred in a health facility. Data from prior births or from those outside of a facility were excluded (figure 1). This resulted in a total of 12 872 eligible mothers included for analysis.

### Definitions

Our main outcome is the women’s report of receiving immediate postnatal health check by a healthcare professional within 24 hours of childbirth while still in the healthcare facility. This was a binary outcome (yes/no). This variable was created with a conceptual link to the WHO Postnatal care recommendations, which state that all women giving birth in healthcare facilities and their babies should remain in the health facility for a minimum of 24 hours following uncomplicated vaginal childbirth, and receive frequent routine postnatal care checks during this period.^15^

We used four variables to construct this outcome, based on separate questions that women were asked: (1) whether the woman received a postnatal check while still in the facility; (2) length of stay of woman in the facility where the birth took place; (3) timing of the first postnatal check in the facility where birth took place and (4) cadre of professional conducting the first postnatal check on mother. As per the WHO recommendations, we would expect optimal immediate postnatal care to be 100% coverage.

There were no differences in the question wording used in the three surveys. Women who reported a stay in the facility of under 24 hours after childbirth needed to have received such a check before discharge. Among women who remained at the facility for 24 hours or more, we used the timing of the postnatal check variable to determine whether the first postnatal health check occurred within 24 hours of childbirth. We categorised health professionals as: doctor, nurse/midwife and medical assistant/clinical officer. To analyse the timing of the postnatal checks within 24 hours among those who received one, we used the women’s response to the question on timing of the first postnatal check in the facility where birth took place and constructed the following categories: (1)<1 hours; (2) 1–4 hours; (3) 4–8 hours; (4) 8–12 hours and (5) 12–24 hours.

We were additionally interested in the women’s report of whether babies delivered in health facilities received a postnatal health check by a health care professional within 24 hours of childbirth while still in the healthcare facility or not. This was done to examine missed opportunity where one of the dyad is checked but not the other. To construct this outcome, we used a composite of four variables akin to the process of constructing the maternal receipt of postnatal care outcome.

### Conceptual framework

**Figure 2:**
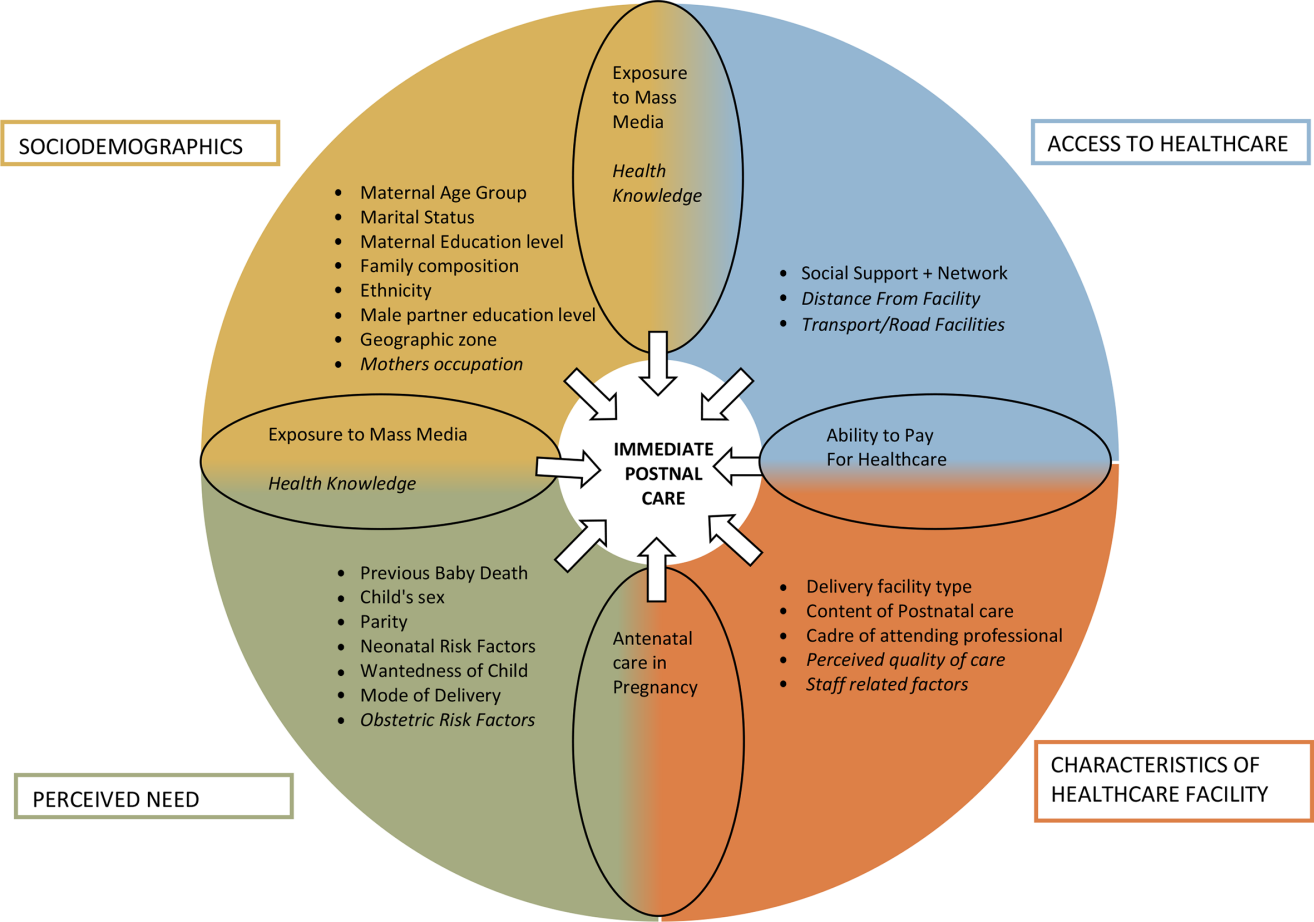
Conceptual framework displaying key factors thought to influence coverage of immediate postnatal care.

We reviewed existing published literature to form a list of key variables noted to impact coverage of postnatal care to create our conceptual framework (figure 2). These were grouped into four categories (socio-demographic factors, access to healthcare, perceived need, and characteristics of healthcare facility). Some factors fell into multiple conceptual categories which have been highlighted in the shaded intersecting portions of the diagram. Unfortunately for certain factors, there were no matching questions asked in the DHS questionnaire and therefore these factors remained in our framework but were not examined in analysis (these are in italics).

#### Perceived need

Seven dimensions of perceived need for postnatal care were identified, including wantedness of child (wanted at the time of pregnancy or not), mode of delivery (vaginal or caesarean), and child sex (female, male). Parity was categorised as first birth, 2–3, 4–5 and 6+births. We were not able to include obstetric or neonatal risk factors (eg, maternal comorbidities and fetal abnormalities) because the DHS did not collect data pertaining to these dimensions. For obstetric risk factors, we considered using proxies such as maternal body mass index and anaemia, however, these data related to the woman at time of the survey and not at time of birth. We, therefore, chose not to include these proxies. For neonatal risk factors, we used the proxy perceived size of the baby at birth which was categorised as very small or not. This was reflective of the perceived need for care by mother as babies perceived to be smaller by the mother are more likely to be seen to and checked. For the dimension ‘death of a previous baby’, we constructed a new variable for previous baby death (no child death, child death within 24 hours of birth, child death later than 24 hours of birth). This variable was explored through sensitivity subgroup analysis of women with previous children (parity >1).

Antenatal care (ANC) in pregnancy was thought to reflect both perceived need and characteristics of healthcare facility. As those women who received facility based ANC were likely to give birth in that same facility. This dimension was examined by categorising the number of ANC visits during the pregnancy (no ANC, 1–3 visits, 4+visits).

Health knowledge and exposure to mass media were thought to reflect the perceived need and sociodemographic factors. There were no questions in the DHS that assessed health knowledge and this dimension could therefore not be analysed further. Exposure to mass media was explored through the variables; any use of television, internet, newspaper and radio (or not) at the time of the survey.

#### Sociodemographic factors

We considered eight sociodemographic factors for inclusion into the model. Maternal age group at birth of baby (in 5-year age groups), marital status (married or cohabiting at time of survey or not), highest maternal education level (no education, primary education, secondary and higher education) and ethnicity (Christian, other religions) were assessed. The boundaries of districts and regions changed over the ten-year period covered by the three DHS and were not identical. We, therefore, constructed four larger zones (Eastern, Western, Northern, Central—see online supplemental material 1) which are consistent over time, as done previously.^6^

10.1136/bmjgh-2020-004230.supp1 Supplementary data

Family composition was assessed by number of persons (<4, 4–5, 6+ persons) and number of children under the age of 5 years (0–1, 2–3, 4+) in the woman’s household. Women’s occupation was not examined, as data in DHS pertained to the time of the survey and not at the time of index birth. Household wealth quintile, place of residence (urban vs rural) and the woman’s autonomy were thought to reflect both socio-demographic factors and access to healthcare factors. Household wealth quintiles were provided in the dataset and constructed using principal component analysis of household assets using an established method.^6^ The dimension of financial autonomy was explored with the binary variable of the woman having a bank account or not. Further exploration of autonomy to healthcare and finances was conducted in sensitivity analysis among married women through the variables who makes decisions about healthcare and finances (respondent alone, respondent and male partner, male partner alone, other). Male partner’s highest education level (no education, primary education, secondary/higher education) was explored further in subgroup analysis among women married at the time of survey.

#### Characteristics of healthcare facility

We identified five dimensions related to characteristics of the healthcare facility where the birth occurred. We categorised the sector of the facility as public (government hospital, government health centre, other public sector) or private (private hospital/clinic, other private medical sector). Assistance with the birth was captured by considering the highest cadre listed (doctor/non-physician clinicians, nurse/midwife, other/none). Staff-related factors were conceptually important, but not available on DHS. The dimension patient perceived quality of care was not directly asked within the DHS and no proxies for this dimension could be found. There were no direct variables that asked women to recall the content of their postnatal care. We used the variable of whether the woman reported that the baby was weighed (or not) as a proxy for this dimension as it is reflective of the available staffing, procedures and resources.

#### Access to healthcare

We were able to assess one dimension—social support and network—for access to healthcare. This dimension was captured by the two created variables: number of persons (<4, 4–5, 6+ persons) and number of children under the age of 5 in the woman’s household (0–1, 2–3, 4+). The distance of the house to the nearest facility or the facility where the birth occurred, or transport/road facilities are not captured on the DHS. Ability to pay for healthcare was thought to reflect both characteristics of healthcare facility and access to healthcare. The variable of whether the woman was covered by health insurance or not was used to reflect this dimension.

### Statistical analysis

All analyses were conducted in STATA V.16 SE. Analysis included descriptive statistics of demographic characteristics of women who gave birth in health facilities on all three surveys. Among women who gave birth in health facilities, we computed the percentage who reported receiving immediate maternal postnatal care. Among women with such a check, we described the distribution of the timing of the first check. We calculated the percentage of babies born in health facilities receiving a postnatal check within 24 hours, disaggregated by type of facility.

For the 2016 survey, we conducted an analysis of mother–baby dyads and calculated the percentage receiving immediate postnatal care within 24 hours while still in healthcare facilities for mother only, baby only, both and neither. Additionally, we used logistic regression to explore the crude associations between factors outlined in the conceptual framework and the woman’s receipt of immediate postnatal care by a health professional in the facility. A multivariable logistic regression model was created by analysing each individual variable and excluding those that were collinear with existing variables. This enabled the multivariable model to be a reflection of the conceptual model.

Two sensitivity analyses using crude and multivariable logistic regression were conducted. First, among the subsample of women married/cohabiting at the time of survey, we additionally included highest level of male partner education, and autonomy with finances and healthcare. Among women with previous children, the model included previous baby death.

We used the survey set command to adjust all analyses for survey sampling design and non-response using individual sampling weights, stratification and clustering.

### Missing data

There were low levels of missing data in the variables used. We describe how missing values were handled in online supplemental material 2.

10.1136/bmjgh-2020-004230.supp2 Supplementary data

It was not appropriate or possible to involve patients or the public in the design, or conduct, or reporting, or dissemination plans of our research.

## Results

### Description of women delivering in healthcare facilities 2006–2016

The sample of women who gave birth in health facilities was 2222 (2006), 3030 (2011) and 7620 (2016). The percentage of most recent births in healthcare facilities increased from 44.6% (95% CI: 41.9% to 47.3%) in 2006 to 60.1% (95% CI: 57.2% to 62.9%) in 2011 to 75.2% (95% CI: 73.4% to 77.0%) in 2016.

Sociodemographic characteristics of women who gave birth in health facilities on each survey are shown in table 1. The majority of births in healthcare facilities were in the public sector on all three surveys.

**Table 1:** Description of women who gave birth in healthcare facilities for most recent live birth in Uganda, DHS 2006, 2010, 2016

Factor	Categories	2006			2011			2016		
		n=2222	%	95% CI	n=3030	%	95% CI	n=7620	%	95% CI
Health facility	Public sector	1563	71.8	68.6 to 74.9	2296	76.1	73.3 to 78.8	6132	78.2	76.4 to 79.9
	Private sector	659	28.2	25.1 to 31.4	734	23.9	21.2 to 26.7	1488	21.8	20.1 to 23.6
Residence	Rural	1679	75.6	70.1 to 80.4	1960	75.6	70.4 to 80.2	5788	72.7	68.5 to 76.5
	Urban	543	24.4	19.6 to 29.9	1070	24.4	19.8 to 29.6	1832	27.3	23.5 to 31.5
Household wealth quintile	Poorest	327	13.9	11.7 to 16.5	476	15.5	13.2 to 18.1	1697	18.2	16.6 to 19.9
	Poorer	362	16.3	14.2 to 18.6	515	17.9	16.0 to 19.8	1457	17.9	16.5 to 19.3
	Middle	341	16.0	14.0 to 18.1	483	17.5	15.7 to 19.5	1389	18.1	16.7 to 19.5
	Richer	458	21.4	18.9 to 24.0	519	18.3	16.3 to 20.6	1392	19.3	17.9 to 21.0
	Richest	734	32.4	28.4 to 36.6	1037	30.8	27.0 to 34.9	1685	26.5	23.6 to 29.6
Geographic zone at survey	Central	784	34.6	31.2 to 38.2	1027	33.8	30.5 to 37.3	1801	30.0	27.4 to 32.7
	Eastern	579	26.5	23.3 to 30.0	682	26.5	23.4 to 29.8	1995	25.6	23.4 to 27.8
	Northern	488	17.7	14.8 to 21.2	767	16.6	14.4 to 19.1	1977	21.1	19.4 to 22.8
	Western	371	21.2	17.9 to 24.9	554	23.1	19.8 to 26.8	1847	23.3	21.8 to 25.1
Maternal age at birth	<20	409	18.5	16.8 to 20.4	493	15.9	14.4 to 17.4	1328	17.4	16.4 to 18.4
	20–24.9	635	29.1	26.9 to 31.4	870	28.7	27.0 to 30.5	2218	29.6	28.3 to 31.0
	25–29.9	566	25.1	23.2 to 27.0	739	24.7	22.9 to 26.6	1826	24.3	23.1 to 25.5
	30–34.9	331	14.8	13.2 to 16.6	482	15.5	14.0 to 17.2	1193	15.0	14.1 to 16.0
	35–49.9	281	12.5	11.1 to 14.0	446	15.2	13.8 to 16.8	1055	13.7	12.8 to 14.6
Highest level of maternal education at survey	No education	299	13.2	11.2 to 15.4	321	9.6	8.2 to 11.3	814	8.8	7.82 to 9.9
	Primary	1304	59.1	56.6 to 61.5	1680	57.7	55.0 to 60.4	4395	55.8	53.7 to 57.8
	Secondary+higher	619	27.7	25.1 to 30.5	1029	32.7	29.8 to 35.6	2411	35.4	33.3 to 37.6
Parity	1	514	23.7	21.8 to 25.7	623	19.4	17.7 to 21.3	1731	23.3	22.1 to 24.5
	2–3	666	29.7	27.6 to 32.0	992	32.3	30.2 to 34.5	2653	35.9	34.5 to 27.3
	4–5	487	21.8	19.9 to 23.7	654	21.3	19.5 to 23.1	1614	20.5	19.6 to 21.6
	6+	555	24.8	22.9 to 26.8	761	27.0	25.0 to 29.1	1622	20.3	19.0 to 21.6
Mode of delivery for last birth	Caesarean birth	178	8.1	6.9 to 9.5	331	10.2	8.9 to 11.7	682	9.7	8.9 to 10.6
	Vaginal birth	2044	91.9	90.5 to 93.1	2699	89.8	88.3 to 91.1	6938	90.3	89.4 to 91.1
Highest cadre of health professional at birth	Doctor/NPC	292	13.1	11.5 to 14.8	486	15.3	13.6 to 17.0	1166	15.8	14.7 to 16.9
	Nurse/midwife	1895	85.5	83.6 to 87.1	2480	82.2	80.4 to 84.0	6321	82.5	81.3 to 83.6
	Non-skilled/other/none	35	1.4	1.0 to 2.2	64	2.50	1.8 to 3.6	133	1.7	1.4 to 2.2

NPC, non-physician clinician (includes medical assistant, clinical officer).

### Receipt of immediate maternal postnatal care

Table 2 reflects the receipt of immediate maternal postnatal care in women who had a health facility birth. Among women who gave birth in healthcare facilities, the percentage that reported receiving an immediate postnatal check increased from 35.7% (95% CI 33.4% to 38.1%) in 2006 to 46.6% in 2011 to 65.0% (95% CI: 63.2% to 66.7%) in 2016. These increases occurred across all social and demographic groups of women. However, in 2016, wide variations in receiving immediate maternal postnatal care remained, particularly in the Western zone, which was far lower than the other zones, and lower among women who had a vaginal birth compared with those with a caesarean section.

**Table 2:** Percentage of women delivering in healthcare facilities who reported receiving immediate postnatal care in Uganda DHS 2006, 2011, 2016

Factor	Categories	2006			2011			2016		
		n	%	95% CI	n	%	95% CI	n	%	95% CI
Overall		805	35.7	33.4 to 38.1	1456	46.6	44.0 to 49.3	4997	65.0	63.2 to 66.7
Health facility	Public sector	555	35.3	32.7 to 38.0	1110	47.0	44.1 to 49.9	4005	64.4	62.6 to 66.2
	Private sector	250	36.6	32.2 to 41.3	734	45.5	40.2 to 50.9	992	66.9	63.4 to 70.2
Residence	Rural	574	33.9	31.3 to 36.6	8731	44.0	40.9 to 47.1	3720	63.0	60.9 to 65.1
	Urban	231	41.2	35.7 to 46.9	585	54.7	48.9 to 60.4	1277	70.2	66.9 to 73.3
Household wealth quintile	Poorest	111	32.9	26.3 to 40.2	234	47.5	41.7 to 53.3	1151	65.7	61.6 to 69.6
	Poorer	104	28.8	23.5 to 34.8	203	39.3	34.2 to 44.6	926	61.6	58.1 to 64.9
	Middle	103	29.9	24.7 to 35.8	199	38.3	33.2 to 43.7	842	59.8	56.4 to 63.0
	Richer	155	32.7	28.3 to 37.4	238	45.5	40.7 to 50.4	854	61.7	58.2 to 65.1
	Richest	332	45.2	41.1 to 49.4	582	55.9	51.1 to 60.1	1224	72.7	65.0 to 75.6
Geographic zone at survey	Central	329	42.1	38.4 to 45.9	581	56.4	52.1 to 60.6	1268	69.9	66.5 to 73.1
	Eastern	222	40.1	35.7 to 44.8	306	46.7	42.0 to 51.4	1413	69.0	65.1 to 72.6
	Northern	168	31.1	25.2 to 37.6	374	46.3	41.6 to 51.1	1378	68.6	64.6 to 72.4
	Western	86	23.5	19.4 to 28.1	195	32.5	27.5 to 37.9	938	50.9	47.9 to 53.9
Maternal age at birth	<20	133	34.3	29.4 to 39.5	238	45.7	40.8 to 50.7	822	61.0	57.9 to 64.1
	20–24.9	221	32.8	29.0 to 36.9	425	46.8	42.4 to 51.3	1440	64.9	62.0 to 67.7
	25–29.9	203	35.1	30.9 to 39.5	341	45.2	40.5 to 49.9	1224	65.7	62.9 to 68.4
	30–34.9	136	40.3	34.9 to 45.9	232	47.6	42.6 to 52.7	815	67.5	63.9 to 70.9
	35–49.9	112	40.1	34.0 to 38.1	220	48.7	43.1 to 54.3	696	65.9	62.4 to 69.3
Highest level of maternal education at survey	No education	92	30.3	25.0 to 36.3	152	41.5	35.4 to 47.9	558	64.1	59.4 to 68.6
	Primary	415	31.5	28.8 to 34.4	728	42.6	39.7 to 45.6	2726	61.3	59.2 to 63.3
	Secondary+higher	298	47.0	42.6 to 51.5	576	55.3	50.4 to 60.0	1713	71.0	68.6 to 73.3
Parity	1	185	36.7	32.2 to 41.4	311	45.7	40.6 to 51.0	1162	66.9	63.9 to 69.7
	2–3	262	38.0	33.7 to 42.5	522	52.3	47.9 to 56.7	1722	64.4	62.0 to 66.8
	4–5	158	31.5	27.3 to 36.0	288	42.8	38.5 to 47.2	1072	65.6	62.5 to 68.6
	6+	200	35.7	31.2 to 40.4	335	43.5	39.6 to 47.4	1041	63.1	60.0 to 66.2
Mode of delivery for last birth	Caesarean birth	101	55.6	46.6 to 64.3	199	61.6	54.8 to 68.1	565	82.2	78.4 to 85.4
	Vaginal birth	704	33.9	31.5 to 36.4	1257	44.9	42.2 to 47.7	4432	63.1	61.3 to 64.9
Highest cadre of health professional at birth	Doctor/NPC	136	45.3	38.5 to 52.2	283	59.8	54.4 to 65.0	853	73.3	69.8 to 76.4
	Nurse/midwife	661	34.4	32.0 to 37.0	1154	44.9	42.1 to 47.8	4072	63.6	61.8 to 65.4
	Non- skilled/other/none	8	22.8	10.1 to 43.7	19	22.7	13.4 to 35.8	72	52.9	42.9 to 62.8

NPC, non-physician clinician.

In 2016, 67.6% (95% CI: 65.8% to 69.3%) of babies born in healthcare facilities received immediate postnatal care. Among the 7620 mother–baby dyads, in 57.9% both the woman and the baby received immediate postnatal care, in 25.4% neither the mother nor the baby received immediate postnatal care, in 7.1% only the mother and in 9.6% only the baby received immediate postnatal care.

### Timing of first maternal postnatal check in healthcare facilities on 2006, 2011, 2016 surveys

Table 3 presents the timing of first maternal postnatal check in healthcare facilities. Across the three surveys, among women who received a postnatal check within 24 hours, the most common time period for the first postnatal check was 1–4 hours after birth; this level was similar in facilities from both sectors. The overall mean time for the first postnatal check decreased from 7.3 hours after birth in 2006 to 4.5 hours in 2011 to 3.1 hours in 2016; and the median time from 4 hours to 2 hours to 1 hour.

**Table 3:** Distribution, mean and median time of first postnatal check in women having immediate postnatal care in healthcare facilities by sector in Uganda DHS 2006, 2011, 2016

	Categories	n	Overall	Public sector	Private sector
2006	<1 hour	90	11.0%	10.8%	11.5%
	1–4 hours	352	44.1%	42.9%	46.6%
	5–8 hours	150	18.6%	19.0%	17.7%
	9–12 hours	59	7.2%	7.8%	5.9%
	13–24 hours	154	19.1%	19.5%	18.3%
	Distribution in hours		Mean (median)	Mean (median)	Mean (median)
	Total	805	7.25 (4)	7.36 (4)	6.99 (3)
2011	<1 hour	261	18.9%	16.4%	27.1%
	1–4 hours	756	52.6%	53.7%	49.1%
	5–8 hours	213	14.3%	15.1%	11.8%
	9–12 hours	89	6.0%	6.8%	3.4%
	13–24 hours	137	8.2%	8.0%	8.6%
	Distribution in hours		Mean (median)	Mean (median)	Mean (median)
	Total	1456	4.49 (2)	4.61 (2)	4.11 (2)
2016	<1 hour	1565	31.8%	30.0%	38.4%
	1–4 hours	2520	49.5%	50.1%	47.5%
	5–8 hours	513	10.3%	11.1%	7.4%
	9–12 hours	190	3.7%	3.8%	3.1%
	13–24 hours	209	4.7%	5.0%	3.6%
	Distribution in hours		Mean (median)	Mean (median)	Mean (median)
	Total	4997	3.09 (1)	3.23 (1)	2.62 (1)

### Factors associated with immediate maternal postnatal check on the 2016 survey

Table 4 presents the association of key factors with receiving an immediate maternal postnatal check in the most recent survey.

**Table 4:** Bivariate and multivariable logistic analysis of factors of coverage of immediate maternal postnatal care of woman who gave birth in healthcare facilities for most recent live births in Uganda 2016

Factor	Categories	Crude analysis (n=7620)			Multivariable analysis (n=7620)		
		OR	95% CI	Wald p-value	aOR	95% CI	Wald p value
Health facility	Public sector	1 (REF)			1 (REF)		
	Private sector	1.12	0.96 to 1.30	0.156	1.14	0.98 to 1.33	0.099
Residence	Rural	0.72	0.60 to 0.87	0.001	0.96	0.78 to 1.17	0.684
	Urban	1 (REF)			1 (REF)		
Household wealth quintile	Poorest	1 (REF)			1 (REF)		
	Poorer	0.84	0.68 to 1.02	0.083	0.95	0.77 to 1.18	0.653
	Middle	0.77	0.61 to 0.98	0.033	0.95	0.74 to 1.22	0.688
	Richer	0.84	0.68 to 1.05	0.125	0.87	0.68 to 1.13	0.300
	Richest	1.39	1.10 to 1.76	0.006	0.98	0.73 to 1.31	0.880
Geographical zone at survey	Central	1.04	0.83 to 1.32	0.718	0.77	0.61 to 0.97	0.024
	Eastern	1 (REF)			1 (REF)		
	Western	0.47	0.38 to 0.58	0.000	0.40	0.32 to 0.49	0.000
	Northern	0.98	0.76 to 1.27	0.895	0.93	0.72 to 1.19	0.549
Maternal age at birth	<20	1 (REF)			1 (REF)		
	20–24.9	1.18	1.00 to 1.38	0.039	1.18	0.98 to 1.42	0.079
	25–29.9	1.22	1.04 to 1.44	0.018	1.24	1.00 to 1.53	0.046
	30–34.9	1.32	1.09 to 1.61	0.005	1.52	1.17 to 1.98	0.002
	35–49.9	1.24	1.01 to 1.51	0.041	1.54	1.15 to 2.06	0.004
Highest level of maternal education at survey	No education	1 (REF)			1 (REF)		
	Primary	0.91	0.73 to 1.13	0.379	0.89	0.73 to 1.08	0.235
	Secondary +higher	1.28	1.03 to 1.61	0.029	0.94	0.74 to 1.18	0.582
Marital status at time of survey	Yes	1.08	0.94 to 1.23	0.260	1.04	0.91 to 1.20	0.553
	No not in union	1 (REF)			1 (REF)		
Total persons in residence at time of survey	<4	1 (REF)					
	four to 5	0.93	0.79 to 1.09	0.379			
	6+	0.88	0.74 to 1.04	0.139			
Total children<5 in residence at time of survey	0–1	1 (REF)					
	2–3	0.99	0.88 to 1.11	0.901			
	4+	0.77	0.58 to 1.03	0.075			
Religion	Christian	0.85	0.71 to 1.01	0.078			
	Other	1 (REF)					
How much of a problem does getting permission pose to getting to the doctor when sick?	Big problem	0.85	0.68 to 1.07	0.160			
	Not a big problem	1.00					
Bank account at time of survey?	Yes	1.77	1.46 to 2.15	0.000	1.26	1.03 to 1.54	0.027
	No	1 (REF)			1 (REF)		
Health insurance at time of survey?	Yes	1.24	0.75 to 2.07	0.398	0.83	0.47 to 1.46	0.521
	No	1 (REF)			1 (REF)		
No of ANC attendances at healthcare facilities	None	1 (REF)			1 (REF)		
	1–3 visits	2.43	1.55 to 3.83	0.000	2.18	1.39 to 3.42	0.001
	4+ visits	2.74	1.74 to 4.31	0.000	2.34	1.50 to 3.64	0.000
Read a newspaper at time of survey	Yes	1.89	1.60 to 2.22	0.000	1.38	1.15 to 1.65	0.000
	Not at all	1 (REF)			1 (REF)		
Listened to the radio at time of survey	Yes	1.22	1.08 to 1.43	0.002	1.13	0.98 to 1.31	0.091
	Not at all	1 (REF)			1 (REF)		
Watched TV at time of survey	Yes	1.49	1.28 to 1.72	0.000			
	Not at all	1 (REF)					
Use of internet at time of survey	Yes	2.40	1.88 to 3.08	0.000	1.39	1.04 to 1.86	0.027
	Not at all	1 (REF)			1 (REF)		
Mobile phone at time of survey	Yes	1.24	1.10 to 1.41	0.000	0.96	0.84 to 1.09	0.501
	No	1 (REF)			1 (REF)		
Parity	One	1 (REF)			1 (REF)		
	Two-three	0.89	0.77 to 1.04	0.140	0.83	0.70 to 0.99	0.033
	Four-five	0.95	0.79 to 1.13	0.547	0.83	0.66 to 1.04	0.110
	Six+	0.85	0.71 to 1.01	0.068	0.75	0.57 to 0.98	0.036
Wantedness of last pregnancy at time of pregnancy	Wanted	1 (REF)			1 (REF)		
	Unwanted	0.85	0.76 to 0.95	0.003	0.87	0.78 to 0.98	0.019
Sex of last baby	Female	1.06	0.95 to 1.20	0.255	1.10	0.98 to 1.24	0.116
	Male	1 (REF)			1 (REF)		
Was baby weighed at birth	Yes	2.08	1.79 to 2.42	0.000	1.84	1.58 to 2.14	0.000
	No	1 (REF)			1 (REF)		
How big did the woman think the baby was at birth	Other	1 (REF)					
	Very small	0.87	0.68 to 1.11	0.259			
Start time of breast feeding	Within an hour	1 (REF)			1 (REF)		
	>1 hours	0.95	0.83 to 1.09	0.474	0.72	0.62 to 0.83	0.000
	Yes-CS	2.70	2.13 to 3.41	0.000	2.93	2.28 to 3.75	0.000
Mode of delivery for last birth	No-vaginal	1 (REF)			1 (REF)		
Highest cadre of health professional at birth	Doctor/NPC	1.56	1.32 to 1.84	0.000			
	Nurse/midwife	1 (REF)					
	Other/none	0.64	0.43 to 0.96	0.03			

ANC, antenatal care; aOR, adjusted OR; CS, Caesarean section; NPC, non-physician clinician; TV, television.

In the multivariable analysis, compared with receiving no facility-based ANC, those with 1–3 visits had an OR of 2.18 (95% CI: 1.39 to 4.2) and those with 4+ visits had an OR 2.34 (95% CI: 1.50 to 3.64) of receiving immediate maternal postnatal care. The use of internet had an OR of 1.39 (95% CI: 1.04 to 1.86) of receiving immediate postnatal care compared with not using the internet. Having the baby weighed at birth had an OR of 1.84 (95% CI: 1.58 to 2.14) of receiving immediate postnatal care compared with not being weighed at birth. Mode of delivery was also an important factor; women who had a caesarean section were 2.93 times (95% CI: 2.28 to 3.75) more likely to receive immediate postnatal care compared with those who had a vaginal birth in a health facility.

### Additional analyses

Within a subsample of 6211 women who were married or cohabiting at the time of the survey (online supplemental material 3), we found in multivariable analysis that male partner’s education was not significantly associated with receipt of immediate postnatal care among women. We analysed a separate sub-sample of 5889 women who had a previous child (online supplemental material 3). In multivariable analysis, women who reported a previous child dying within 24 hours of birth were not more likely to have reported an immediate maternal postnatal check than women without a previous child death (adjusted OR 1.05 (95% CI: 0.80 to 1.38))

10.1136/bmjgh-2020-004230.supp3 Supplementary data

## Discussion

Over the 15-year period under investigation, when the percentage of births in health facilities increased, the percentage of women who remained in health facilities for 24 hours or longer remained stable at just above 70%, and coverage of immediate maternal postnatal care after facility birth increased to 65.0%. We found large geographic variations in immediate maternal postnatal care after facility births on the 2016 survey, particularly between Western and Eastern Uganda. In the 2016 survey, the proportion of babies and mothers receiving immediate postnatal care was similar. The majority of first postnatal checks after facility births occurred at 1–4 hours post partum and the median time interval from birth to first check reduced between 2006 and 2016 surveys, with the shift towards earlier checks most marked in private sector facilities. The most significant factor independently associated with receipt of immediate postnatal care was mode of delivery; women who had a caesarean section had nearly three times the odds of immediate postnatal care compared with those with a vaginal birth. Other factors positively associated with higher odds of being checked included exposure to mass media, baby having been weighed at birth, and receipt of ANC.

Our findings show the most significant improvement to postnatal care provision occurred between 2011 and 2016 where coverage increased by 20%. This is in line with the 15.1% increase in deliveries in healthcare facilities in line with current global trends and recommendations. Despite this, immediate postnatal care coverage after such births remained suboptimal at 65%.^15 27^ This rate is higher than the 50% coverage reported in Uganda by Ndugga *et al*, although this report included births at both home and facility.^28^ A common reason given for poor coverage of care is that women do not remain in facilities long enough (for 24 hours) to receive postnatal care.^16^ However, our study shows that even if women do stay for 24 hours, the coverage of immediate postpartum checks still remains suboptimal. As such, this finding may be more reflective of the lack of priority given to mothers in the postpartum period.^29^ At present, coverage of immediate postpartum checks is globally poor and the worst performing aspect in the obstetric continuum of care.^7 8^ In fact, there is no current global standard metric available to evaluate progress in quality and content of care which otherwise exists for both antenatal and intrapartum care.^30^ Additionally, with the increased number of facility births, health facilities are busier than ever before. The poor coverage of immediate care could therefore be a sign of overburdened and burned-out healthcare providers struggling to keep up with the increasing demand.^31^ Furthermore, although WHO Postnatal care recommendations do exist, there is a paucity of literature providing guidance and frameworks to health workers at country or system level on how to provide high quality maternal care, particularly after facility birth.^32^ As a result, the care provided is often incomplete and not universal to every women at birth.^17^

Although not universal, women from Eastern Uganda were noted to receive the highest coverage of immediate postnatal care after facility birth. In a cross-sectional study of factors affecting utilisation of early postnatal care (within 7 days of birth) in Eastern Uganda following health facility births, formal employment was identified as the key socioeconomic factor increasing the likelihood of women receiving early postnatal care.^33^ Through employment, women not only have a better financial status and ability to use quality health services but are also empowered to participate in the decision-making process about their healthcare.^33^ DHS data do not provide the means to accurately examine the role of a women’s occupation on receipt of immediate postnatal care and therefore it might be useful to examine this factor directly at regional level. Interestingly, there was poorer coverage of care in Central Uganda than Eastern Uganda. The Central Uganda region contains Kampala, the capital and largest city in Uganda. Coupled with the rise in health facility births, crowding does exist within facilities and this has been found to have an ongoing detrimental effect on the quality of respectful care received by women.^8 31^ Other examples of a lack of respectful postnatal care in Uganda (physical and verbal mistreatment by staff, and stigma and denial of care for marginalised communities) have been shown to contribute to a negative care experience by mothers.^34^ This has resulted in a reduced utilisation and therefore coverage of postnatal care services.^35^ Again, data for respectful care were not collected in the DHS and this would also be worth examining directly at regional level.

Conceptually, a key factor thought to impact maternal immediate postnatal care provision is if immediate postnatal care is provided for the baby. In the 2016 survey, nearly two-thirds of mother–baby dyads received both maternal and neonatal immediate postnatal care, while a quarter did not receive any immediate postnatal care for either mother or baby. Very few women reported receiving only maternal or only neonatal immediate postnatal care. This suggests that if a baby is to receive immediate postnatal care, a mother is likely to as well, and vice versa. This finding is perhaps driven by the notion that those mothers and babies with complications (eg, mothers most unwell following birth or with existing conditions, and babies which are smaller, premature or who are most unwell), will be prioritised to receive care.^34^ This idea is further exemplified in our multivariable model which found reporting that the baby was weighed at birth was positively associated with the mother received an immediate postnatal check. To our knowledge, there is no existing literature examining this interaction. With this knowledge, further work perhaps should focus on integrating maternal and newborn immediate postnatal care within in-facility postnatal care guidelines to aid in improving coverage of overall immediate postnatal care.^8^

The timing of the first postnatal check, examined in all datasets, found that the majority of first checks occurred consistently between 1 and 4 hours. This is in line with Ugandan guidelines and several factors encourage this early postnatal review.^22^ For example, many resource-stretched maternity units are in need of early discharge to make space for new deliveries.^16^ Additionally, for women delivering in private facilities, having an early check will often enable women and babies to leave as soon as possible to reduce the cost incurred for time spent in the facility.^16 36^ Unfortunately, the DHS did not gather data on quality or components of postnatal checks, and it was not possible to determine if the speed of check compromises the quality of care provided. Early postnatal checks are not necessarily ideal.^16^ WHO recommends that all new mothers remain in the facility for 24 hours following birth because the majority of maternal and neonatal deaths occur during this period.^9^ Mothers receiving only one check in the first hour following delivery therefore, without any further checks, might not be sufficiently monitored to detect complications of birth.^9 15^ As such, it has been suggested that in sub-Saharan Africa >75% of women receive suboptimal postnatal health checks.^16 17^ Further qualitative work focusing on quality of care and actions taken following postnatal checks would be important to enable this finding to be explored further.

Factors affecting coverage of immediate postnatal care were explored through crude and multivariable logistic regression analysis in the 2016 database. Having a caesarean section increased the odds of being checked compared with those women who had a vaginal delivery which has been noted in existing literature from Bangladesh.^37^ Women having a caesarean section are at a higher risk of severe acute maternal morbidity, particularly in low-resource settings.^38^ As such, clinicians faced with time pressures and a large number of patients to see, might prioritise examining high risk postoperative women.^17^ That said, coverage of immediate postnatal care for mothers giving birth by caesarean section was still suboptimal at 82% (2016). It is, therefore, imperative that new strategies are looked into to improve postnatal care coverage. In sub-Saharan Africa, up to 20% of caesarean sections are conducted under general anaesthesia.^39^ Following a long labour and emergency caesarean under general anaesthesia, women can often be drowsy and disorientated resulting in poor recall of birth and postnatal care events. The figure of 82% coverage should, therefore, be interpreted with caution.

Women who had access to mass media were noted to have a higher likelihood of receiving immediate postnatal care. This finding is in line with previous studies across sub-Saharan Africa.^40^ Mass media can be harnessed as a platform to educate and inform mothers in order increase their access to knowledge and improve their ability to seek care.^28^ In all low-resource settings, education has consistently been noted as key to postnatal care utilisation.^41^ Additionally, coverage of ANC was identified as an important factor. The more antenatal visits a woman received the higher the chance of receiving immediate maternal postnatal care compared with those receiving no ANC in facility. This finding was in line with studies from Ethiopia.^42^ ANC has also been proven a worthy platform to provide effective maternal education to reduce postpartum morbidity and may provide the opportunity for mothers to understand key features of their continuum of care.^43^ This finding could be confounded by wealth/social capital as well as the presence of any pre-existing conditions requiring receipt of more ANC and therefore resulting in a higher need to be checked postnatally.

### Limitations

By using three DHS datasets from 2006 to 2016, we were able to explore changes over time over 15 years of births based on recall periods and examine the coverage and determinants of coverage of immediate postnatal care in health facilities. There are, however, some limitations to our work. First, the DHS relies on women’s recall of the immediate postnatal period up to 5 years preceding the survey. This relies on the accuracy of the woman’s memory of the receipt and timing of postnatal care provided in the immediate postnatal period. A study in two sub-Saharan African settings showed that women were able to recall key postnatal events to an acceptable threshold.^44^ However, our results flagged some clear anomalies as across the three surveys women consistently reported caesarean sections being conducted by nurses/midwives and unskilled birth attendants. This could be that women are actually remembering the latter parts of intrapartum care prior to birth. Studies on the validity of women’s recall in the immediate postnatal care have found that women are consistently less able to recall indicators in the intrapartum period and within 1 hour of delivery immediately postnatally.^45 46^ With checks being conducted earlier, there is additionally a higher chance of women being unable to distinguish between the latter parts of intrapartum care and immediate postnatal care.^46^ We attempted to limit the extent of recall error by restricting responses to the respondents’ most recent birth. Second, the DHS questions do not capture the presence of complications, nor the quality and content of postnatal care. Our ability to examine postnatal care comprehensively and adjust for important confounders was therefore somewhat limited.^44^ Third, demographic data within the DHS survey such as marital status are taken at the time of the survey being conducted and not at the time of birth. That said, the DHS recall period of live births is limited to 5 years and thus it limits the extent of discrepancies.

## Conclusion

Although there have been significant advances in coverage of postnatal care globally, there still remains a large gap. This article works synergistically with other global papers to strengthen the argument for focus and attention to postnatal care. This is especially relevant and important as it paves the way for new postnatal care guidelines due to be released by WHO in 2021. Future research in postnatal care coverage is key to ensure it gains a pivotal place on the global maternal health agenda. In Uganda, it would be useful to understand the barriers to provision of immediate postnatal care after facility births at national level. This would enable policy stakeholders to galvanise support in prioritising postnatal care provision and equitable coverage. Additionally, it would provide data on how to ensure best practice through guidelines training and implementation. One strategy could be to look at further integrating maternal and newborn care services, even beyond discharge. Another would be to acknowledge that increasing facility-based births or the number of women remaining in facility for 24 hours following birth has not led to the sufficient change needed to make immediate postnatal care coverage universal, nor reduce maternal and neonatal mortality. If anything, the resulting crowding has increased the pressure on overburdened and burned-out healthcare providers, reducing respectful quality postnatal care provision and preventing the swift recognition and action if postnatal complication are identified. Perhaps education and active involvement of mothers and their partners in their care could act to enhance respectful care and improve coverage and utilisation of care. Finally, there is need for a direct needs assessment of health system, staff and quality of care, to identify factors that impede immediate postnatal care coverage. It would be useful for the work to be conducted by region to help uncover the geographical and subnational differences that exist in care provision.

## Data Availability

Data are available in a public, open access repository. The data included in this study is non-identifiable, anonymised data from the publicly available DHS data sets from Uganda. The data sets were analysed during this study and all analysis are presented in the main article and online supplemental material. All data analysis is held by the submitting author (TD ORCID identifier https://orcid.org/0000-0003-1326-5107).

## References

[1] WHO. Trends in maternal mortality 2000 to 2017: estimates by who, UNICEF, UNFPA, world bank group and the United nations population division. Geneva: World Health Organization, 2019.

[2] Hug L, Alexander M, You D, et al. National, regional, and global levels and trends in neonatal mortality between 1990 and 2017, with scenario-based projections to 2030: a systematic analysis. Lancet Glob Health 2019;7:e710–20. 10.1016/S2214-109X(19)30163-9 31097275 PMC6527519

[3] UN. The sustainable development goals report 2020. Geneva: United Nations, 2020. https://unstats.un.org/sdgs/report/2020/The-Sustainable-Development-Goals-Report-2020.pdf

[4] UN. Progress towards the sustainable development goals report of the Secretary-General 2019. Geneva: United Nations, 2019. https://sustainabledevelopment.un.org/content/documents/24978Report_of_the_SG_on_SDG_Progress_2019.pdf

[5] Uganda Bureau of Statistics - UBOS and ICF. 2018. Uganda demographic and health survey. Kampala, Uganda: UBOS and ICF, 2016. http://dhsprogram.com/pubs/pdf/FR333/FR333.pdf

[6] Benova L, Dennis ML, Lange IL, et al. Two decades of antenatal and delivery care in Uganda: a cross-sectional study using demographic and health surveys. BMC Health Serv Res 2018;18:758. 10.1186/s12913-018-3546-3 30286749 PMC6172797

[7] Victora CG, Requejo JH, Barros AJD, et al. Countdown to 2015: a decade of tracking progress for maternal, newborn, and child survival. Lancet 2016;387:2049–59. 10.1016/S0140-6736(15)00519-X 26477328 PMC7613171

[8] Sacks E, Langlois Étienne V. Postnatal care: increasing coverage, equity, and quality. Lancet Glob Health 2016;4:e442–3. 10.1016/S2214-109X(16)30092-4 27185467

[9] Ronsmans C, Graham WJ, Lancet Maternal Survival Series steering group. Maternal mortality: who, when, where, and why. Lancet 2006;368:1189–200. 10.1016/S0140-6736(06)69380-X 17011946

[10] WHO. Technical consultation on postpartum and postnatal care. Geneva: WHO, 2010. https://apps.who.int/iris/bitstream/handle/10665/70432/WHO_MPS_10.03_eng.pdf?sequence=1 26269861

[11] Khan KS, Wojdyla D, Say L, et al. Who analysis of causes of maternal death: a systematic review. Lancet 2006;367:1066–74. 10.1016/S0140-6736(06)68397-9 16581405

[12] Birko S, Dove ES, Özdemir V. Evaluation of nine consensus indices in Delphi foresight research and their dependency on Delphi survey characteristics: a simulation study and debate on Delphi design and interpretation. PLoS One 2015;10:e0135162. 10.1371/journal.pone.0135162 26270647 PMC4535950

[13] GBD 2013 Mortality and Causes of Death Collaborators. Global, regional, and national age-sex specific all-cause and cause-specific mortality for 240 causes of death, 1990-2013: a systematic analysis for the global burden of disease study 2013. Lancet 2015;385:117–71. 10.1016/S0140-6736(14)61682-2 25530442 PMC4340604

[14] Campbell OMR, Graham WJ, Lancet Maternal Survival Series steering group. Strategies for reducing maternal mortality: getting on with what works. Lancet 2006;368:1284–99. 10.1016/S0140-6736(06)69381-1 17027735

[15] WHO. Who recommendations on postnatal care of the mother and newborn. Geneva: WHO, 2019. https://apps.who.int/iris/bitstream/handle/10665/97603/9789241506649_eng.pdf?sequence=1 24624481

[16] Campbell OMR, Cegolon L, Macleod D, et al. Length of stay after childbirth in 92 countries and associated factors in 30 low- and middle-income countries: compilation of reported data and a cross-sectional analysis from nationally representative surveys. PLoS Med 2016;13:e1001972. 10.1371/journal.pmed.1001972 26954561 PMC4783077

[17] Benova L, Owolabi O, Radovich E, et al. Provision of postpartum care to women giving birth in health facilities in sub-Saharan Africa: a cross-sectional study using demographic and health survey data from 33 countries. PLoS Med 2019;16:e1002943. 10.1371/journal.pmed.1002943 31644531 PMC6808422

[18] Munabi-Babigumira S, Nabudere H, Asiimwe D, et al. Implementing the skilled birth attendance strategy in Uganda: a policy analysis. BMC Health Serv Res 2019;19:655. 10.1186/s12913-019-4503-5 31500636 PMC6734264

[19] Konde-Lule J, Gitta SN, Lindfors A, et al. Private and public health care in rural areas of Uganda. BMC Int Health Hum Rights 2010;10:29. 10.1186/1472-698X-10-29 21106099 PMC3003635

[20] Parkhurst JO, Penn-Kekana L, Blaauw D, et al. Health systems factors influencing maternal health services: a four-country comparison. Health Policy 2005;73:127–38. 10.1016/j.healthpol.2004.11.001 15978956

[21] Wallace LJ, Kapiriri L. Priority setting for maternal, newborn and child health in Uganda: a qualitative study evaluating actual practice. BMC Health Serv Res 2019;19:465. 10.1186/s12913-019-4170-6 31286950 PMC6615092

[22] The Republic of Ugandan Ministry of Health. National guidelines for management of common conditions Uganda clinical guidelines, 2016.

[23] de Graft-Johnson J, Vesel L, Rosen HE, et al. Cross-Sectional observational assessment of quality of newborn care immediately after birth in health facilities across six sub-Saharan African countries. BMJ Open 2017;7:e014680. 10.1136/bmjopen-2016-014680 PMC5372100 28348194

[24] Duysburgh E, Kerstens B, Kouanda S, et al. Opportunities to improve postpartum care for mothers and infants: design of context-specific packages of postpartum interventions in rural districts in four sub-Saharan African countries. BMC Pregnancy Childbirth 2015;15:131. 10.1186/s12884-015-0562-8 26038100 PMC4453099

[25] Belemsaga DY, Kouanda S, Goujon A, et al. A review of factors associated with the utilization of healthcare services and strategies for improving postpartum care in Africa. Afrika Focus 2015;28:83–105. 10.1163/2031356X-02802006

[26] Countdown to 2030 Collaboration. Countdown to 2030: tracking progress towards universal coverage for reproductive, maternal, newborn, and child health. Lancet 2018;391:1538–48. 10.1016/S0140-6736(18)30104-1 29395268

[27] Souza JP, Gülmezoglu AM, Vogel J, et al. Moving beyond essential interventions for reduction of maternal mortality (the who multicountry survey on maternal and newborn health): a cross-sectional study. Lancet 2013;381:1747–55. 10.1016/S0140-6736(13)60686-8 23683641

[28] Ndugga P, Namiyonga NK, Sebuwufu D, ogratious SD. Determinants of early postnatal care attendance: analysis of the 2016 Uganda demographic and health survey. BMC Pregnancy Childbirth 2020;20:163. 10.1186/s12884-020-02866-3 32178635 PMC7076947

[29] Dhaher E, Mikolajczyk RT, Maxwell AE, et al. Factors associated with lack of postnatal care among Palestinian women: a cross-sectional study of three clinics in the West bank. BMC Pregnancy Childbirth 2008;8:26. 10.1186/1471-2393-8-26 18638395 PMC2499989

[30] Moran AC, Kerber K, Sitrin D, et al. Measuring coverage in MNCH: indicators for global tracking of newborn care. PLoS Med 2013;10:e1001415. 10.1371/journal.pmed.1001415 23667335 PMC3646209

[31] Weeks A, Lavender T, Nazziwa E, et al. Personal accounts of 'near-miss' maternal mortalities in Kampala, Uganda. BJOG 2005;112:1302–7. 10.1111/j.1471-0528.2005.00703.x 16101612

[32] Sakala B, Chirwa E. An evidence-based policy brief: improving the quality of postnatal care in mothers 48 hours after childbirth. Malawi Med J 2019;31:164–8. 10.4314/mmj.v31i2.12 31452853 PMC6698620

[33] Izudi J, Amongin D. Use of early postnatal care among postpartum women in eastern Uganda. Int J Gynaecol Obstet 2015;129:161–4. 10.1016/j.ijgo.2014.11.017 25661323

[34] Sacks E, Masvawure TB, Atuyambe LM, et al. Postnatal care experiences and barriers to care utilization for Home- and Facility-Delivered newborns in Uganda and Zambia. Matern Child Health J 2017;21:599–606. 10.1007/s10995-016-2144-4 27475823

[35] Sacks E, Kinney MV. Respectful maternal and newborn care: building a common agenda. Reprod Health 2015;12:46. 10.1186/s12978-015-0042-7 25986552 PMC4460639

[36] Ntambue AM, Malonga FK, Dramaix-Wilmet M, et al. Commercialization of obstetric and neonatal care in the Democratic Republic of the Congo: a study of the variability in user fees in Lubumbashi, 2014. PLoS One 2018;13:e0205082. 10.1371/journal.pone.0205082 30304060 PMC6179261

[37] Kim ET, Singh K, Weiss W. Maternal postnatal care in Bangladesh: a closer look at specific content and coverage by different types of providers. J Glob Health Rep 2019;3:e2019004. 10.29392/joghr.3.e2019004 31482138 PMC6720114

[38] Sandall J, Tribe RM, Avery L, et al. Short-Term and long-term effects of caesarean section on the health of women and children. Lancet 2018;392:1349–57. 10.1016/S0140-6736(18)31930-5 30322585

[39] Bishop D, Dyer RA, Maswime S, et al. Maternal and neonatal outcomes after caesarean delivery in the African surgical outcomes study: a 7-day prospective observational cohort study. Lancet Glob Health 2019;7:e513–22. 10.1016/S2214-109X(19)30036-1 30879511

[40] Tessema ZT, Yazachew L, Tesema GA, et al. Determinants of postnatal care utilization in sub-Saharan Africa: a meta and multilevel analysis of data from 36 sub-Saharan countries. Ital J Pediatr 2020;46:175. 10.1186/s13052-020-00944-y 33246475 PMC7693498

[41] Adams YJ, Smith BA. Integrative review of factors that affect the use of postpartum care services in developing countries. J Obstet Gynecol Neonatal Nurs 2018;47:371–84. 10.1016/j.jogn.2018.02.006 29524378

[42] Fekadu GA, Ambaw F, Kidanie SA. Facility delivery and postnatal care services use among mothers who attended four or more antenatal care visits in Ethiopia: further analysis of the 2016 demographic and health survey. BMC Pregnancy Childbirth 2019;19:64. 10.1186/s12884-019-2216-8 30744583 PMC6371418

[43] Bhutta ZA, Das JK, Bahl R, et al. Can available interventions end preventable deaths in mothers, newborn babies, and stillbirths, and at what cost? Lancet 2014;384:347–70. 10.1016/S0140-6736(14)60792-3 24853604

[44] Amouzou A, Mehra V, Carvajal-Aguirre L, et al. Measuring postnatal care contacts for mothers and newborns: an analysis of data from the MICs and DHS surveys. J Glob Health 2017;7:020502. 10.7189/jogh.07.020502 29423179 PMC5804502

[45] McCarthy KJ, Blanc AK, Warren C. Validating women’s reports of antenatal and postnatal care received in Bangladesh, Cambodia and Kenya. BMJ Glob Heal 2020;5:2133. 10.1136/bmjgh-2019-002133

[46] Amouzou A, Hazel E, Vaz L, et al. Discordance in postnatal care between mothers and newborns: measurement artifact or missed opportunity? J Glob Health 2020;10. 10.7189/jogh.10.010505 PMC7101084 32257159

