# Supplementary material for: Immediate postnatal care following childbirth in Ugandan health facilities: an analysis of Demographic and Health Surveys between 2001 and 2016
Source: BMJ Glob Health. 2021 Apr 22;6(4):e004230. doi: 10.1136/bmjgh-2020-004230 (PMC8070850; doi:10.1136/bmjgh-2020-004230)
Supplement: Supplementary data [file bmjgh-2020-004230supp001.pdf]

Supplementary Material 1

Table 1 – DHS districts by zone and survey year

	**DHS districts included in each zone, by survey year**		
**Zone**	**2006**	**2011**	**2016**
**Central**	Central 1, Central 2 and Kampala	Central 1, Central 2 and Kampala	South central, North central, Kampala
**Eastern**	Eastern and East Central	Eastern and East Central	Busoga, Bukedi, Bugisu, Teso,
**Northern**	West Nile and North	West Nile and North and Karamoja	Karamoja, Lango, Acholi, West Nile
**Western**	Western and Southwest	Western and Southwest	Bunyoro, Kigezi, Ankole, Tororo
