# Supplementary material for: Immediate postnatal care following childbirth in Ugandan health facilities: an analysis of Demographic and Health Surveys between 2001 and 2016
Source: BMJ Glob Health. 2021 Apr 22;6(4):e004230. doi: 10.1136/bmjgh-2020-004230 (PMC8070850; doi:10.1136/bmjgh-2020-004230)
Supplement: Supplementary data [file bmjgh-2020-004230supp002.pdf]

Supplementary Material 2

In the 2006 dataset, the mode of delivery component identified 1 missing data point which was recoded as vaginal delivery as it was thought a women likely to remember having a caesarean section if she had had one. For the highest cadre of healthcare worker at delivery, 5 responders selected both doctor and NPC and so they were only counted once. When creating the component of women being checked within 24 hours, 1417 responders were classed as missing which accounted for women who had no check, women who had a check, but the timing recorded was missing as well as women who had a check but >24 hours. We excluded these values.

In the 2011 dataset, for the highest cadre of healthcare worker at delivery, 4 responders selected both doctor and NPC and so they were only counted once. When creating the component of women being checked within 24 hours, 1574 responders were classed as missing which accounted for women who had no check, women who had a check, but the timing recorded was missing as well as women who had a check but >24 hours. We excluded these values.

In the 2016 dataset, the mode of delivery component identified 39 missing data points which was recoded as vaginal delivery as it was thought a women likely to remember having a caesarean section if she had had one. For the highest cadre of healthcare worker at delivery, 22 responders selected both doctor and NPC and so they were only counted once. When creating the component of women being checked within 24 hours, 2623 responders were classed as missing which accounted for women who had no check, women who had a check, but the timing recorded was missing as well as women who had a check but >24 hours. We excluded these values. When creating the component who makes decisions about healthcare and who makes decisions about large household purchases, 1409 missing responses were noted and were counted as women who aren't married/don't have a partner and therefore are excluded from analysis. When creating the component number of facility-based ANC at time of index pregnancy, 37 responses were "don't know" which we have pragmatically classed as '1-3 visits' as it is likely that if the woman had received no ante natal care, she would have reported this. Equally if the woman had received more than 4 visits this would have been reported too. Additionally, 32 women reported having some antenatal care but not in a health facility which we have classed as "0" visits. When creating the previous baby death variable, 1731 responses were missing which were for women who had no previous child and therefore were excluded from the variable. When looking at the was baby weighed at birth variable, there were 128 'don't know' responses which we recoded as not being weighed at birth as otherwise the mother would likely have remembered. For the size of baby variable, 55 responders classed the baby weight as "don't know" which suggested the weight was not significantly small to be noticeable and therefore these responses were classed as "other". For time of breastfeeding, there were 197 missing responses which we recoded as being over 1 hour as it is likely a woman would remember if breastfeeding occurred immediately or within an hour.
