# Supplementary material for: Immediate postnatal care following childbirth in Ugandan health facilities: an analysis of Demographic and Health Surveys between 2001 and 2016
Source: BMJ Glob Health. 2021 Apr 22;6(4):e004230. doi: 10.1136/bmjgh-2020-004230 (PMC8070850; doi:10.1136/bmjgh-2020-004230)
Supplement: Supplementary data [file bmjgh-2020-004230supp003.pdf]

Supplementary Material 3

Crude and Multivariable logistic analysis of a subgroup of married women who received immediate postnatal care in 2016 and subgroup of women who had previous children and who received immediate postnatal care in 2016

		Subgroup Married women- Crude analysis (n=6211)			Subgroup Married women- Multivariable analysis (n=6211)			Subgroup Women with previous children- Crude analysis (n=5889)			Subgroup Women with previous children- Multivariable analysis		
Factor		OR	95% CI	Wald p-value	aOR	95% CI	Wald p-value	OR	95% CI	Wald p-value	aOR	95% CI	Waldp-value
Health facility	Public sector	1 (REF)			1 (REF)			1 (REF)			1 (REF)		
	Private sector	1.12	0.95-1.33	0.173	1.13	0.95-1.35	0.158	1.11	0.94-1.31	0.234	1.12	0.94-1.34	0.188
Residence	Rural	0.72	0.59-0.86	0.001	0.96	0.77-1.20	0.716	0.76	0.62-0.92	0.006	1.01	0.81-1.24	0.960
	Urban	1 (REF)			1 (REF)			1 (REF)			1 (REF)		
Household wealth quintile	Poorest	1 (REF)			1 (REF)			1 (REF)			1 (REF)		
	Poorer	0.82	0.65-1.03	0.094	0.92	0.73-1.18	0.521	0.81	0.64-1.03	0.080	0.93	0.73-1.20	0.598
	Middle	0.81	0.62-1.05	0.112	1.00	0.76-1.32	0.997	0.79	0.60-1.04	0.091	0.96	0.72-1.29	0.811
	Richer	0.86	0.68-1.09	0.201	0.90	0.68-1.18	0.446	0.86	0.67-1.10	0.224	0.91	0.69-1.21	0.537
	Richest	1.47	1.15-1.87	0.002	1.01	0.73-1.40	0.936	1.42	1.08-1.85	0.012	0.96	0.69-1.33	0.809
Geographic zone at survey	Central	1.07	0.84-1.37	0.575	0.73	0.57-0.94	0.013	1.06	0.82-1.37	0.655	0.78	0.60-1.00	0.053
	Eastern	1 (REF)			1 (REF)			1 (REF)			1 (REF)		
	Western	0.47	0.38-0.58	0.000	0.38	0.30-0.48	0.000	0.46	0.36-0.59	0.000	0.40	0.31-0.51	0.000
	Northern	0.99	0.76-1.28	0.932	0.97	0.75-1.26	0.831	1.04	0.79-1.36	0.805	0.99	0.76-1.29	0.940
Maternal age at birth	<20	1 (REF)			1 (REF)			1 (REF)			1 (REF)		
	20-24.9	1.10	0.92-1.32	0.286	1.04	0.84-1.29	0.718	1.38	1.06-1.79	0.016	1.24	0.95-1.63	0.108
	25-29.9	1.26	1.04-1.53	0.019	1.18	0.92-1.52	0.203	1.46	1.11-1.92	0.007	1.21	0.90-1.63	0.211
	30-34.9	1.29	1.03-1.61	0.028	1.42	1.05-1.92	0.024	1.64	1.25-2.14	0	1.49	1.07-2.06	0.017
	35-49.9	1.21	0.96-1.54	0.113	1.49	1.06-2.10	0.023	1.54	1.15-2.06	0.004	1.51	1.05-2.16	0.025
Highest level of maternal education at survey	No education	1 (REF)			1 (REF)			1 (REF)			1 (REF)		
	Primary	0.93	0.75-1.15	0.506	0.94	0.76-1.16	0.547	0.90	0.73-1.10	0.31	0.89	0.73-1.10	0.289
	Secondary+higher	1.42	1.12-1.81	0.004	0.98	0.75-1.26	0.849	1.46	1.16-1.85	0.001	1.00	0.76-1.30	0.975
Highest level of education of husband at survey	No education	1 (REF)			1 (REF)								
	Primary	0.86	0.67-1.11	0.248	0.88	0.69-1.13	0.332						
	Secondary+higher	1.20	0.92-1.56	0.176	0.88	0.67-1.15	0.346						
Marital status at time of survey	Yes							1.21	1.03-1.42	0.020	1.15	0.98-1.35	0.095
	No not in union							1 (REF)			1 (REF)		
Who makes decisions about healthcare	Respondent	0.99	0.43-2.29	0.976	1.42	0.57-3.56	0.448						
	Patient+ husband/partner	1.10	0.48-2.53	0.828	1.77	0.70-4.46	0.223						
	Husband/partner	0.86	0.37-2.00	0.727	1.39	0.56-3.48	0.479						
	Other/ someone else	1 (REF)			1 (REF)								
Who makes decisions on large household purchases	Respondent	0.50	0.18-1.34	0.166	0.32	0.09-1.06	0.063						
	Patient+ husband/partner	0.63	0.24-1.68	0.356	0.37	0.11-1.24	0.107						
	Husband/partner	0.58	0.22-1.57	0.284	0.39	0.12-1.29	0.123						
	Other/ someone else	1 (REF)			1 (REF)								
Bank account at time of survey?	Yes	1.76	1.44-2.15	0..00	1.24	1.00-1.54	0.055	1.75	1.42-2.16	0.000	1.20	0.96-1.51	0.116
	No	1 (REF)			1 (REF)			1 (REF)			1 (REF)		
Health insurance at time of survey	Yes	1.09	0.64-1.85	0.759	0.74	0.40-1.36	0.326	1.19	0.69-2.06	0.526	0.79	0.43-1.48	0.468
	No	1 (REF)			1 (REF)			1 (REF)			1 (REF)		
Number of ANC attendances at health care facilities	0	1 (REF)			1 (REF)			1 (REF)			1 (REF)		
	1-3 visits	1.86	1.07-3.22	0.027	1.68	0.96-2.94	0.069	2.35	1.36-4.06	0.002	2.09	1.22-3.59	0.007
	4+ visits	2.10	1.22-3.61	0.007	1.83	1.06-3.15	0.030	2.60	1.50-4.48	0.001	2.19	1.29-3.74	0.004
Read a newspaper at time of survey	Yes	1.89	1.58-2.26	0.000	1.38	1.14-1.67	0.001	1.86	1.55-2.23	0.000	1.29	1.05-1.58	0.014
	Not at all	1 (REF)			1 (REF)			1 (REF)			1 (REF)		
Listened to the radio at time of survey	Yes	1.28	1.10-1.49	0.001	1.16	0.99-1.36	0.067	1.31	1.12-1.54	0.001	1.21	1.01-1.44	0.035
	Not at all	1 (REF)			1 (REF)			1 (REF)			1 (REF)		
Use of Internet at time of survey	Yes	2.41	1.80-3.22	0.000	1.33	0.94-1.87	0.105	3.44	2.30-5.16	0.000	1.89	1.19-3.02	0.007
	Not at all	1 (REF)			1 (REF)			1 (REF)			1 (REF)		
Mobile phone at time of survey	Yes	1.32	1.15-1.51	0.000	1.01	0.87-1.18	0.888	1.27	1.11-1.46	0.001	1.01	0.87-1.16	0.936
	No	1 (REF)			1 (REF)			1.00	REF	REF	1.00	REF	REF
Parity	One	1 (REF)			1 (REF)					DROPPED			DROPPED
	Two-three	0.95	0.80-1.13	0.572	0.94	0.76-1.16	0.552	1 (REF)			1 (REF)		
	Four-five	1.02	0.83-1.25	0.878	0.96	0.74-1.26	0.778	1.06	0.91-1.22	0.466	1.07	0.89-1.29	0.493
	six+	0.85	0.69-1.04	0.109	0.79	0.57-1.08	0.137	0.95	0.81-1.11	0.488	1.01	0.78-1.30	0.940
Wantedness of last pregnancy at time of pregnancy	Wanted	1 (REF)			1 (REF)			1 (REF)			1 (REF)		
	Unwanted	0.82	0.73-0.92	0.001	0.84	0.74-0.96	0.010	0.79	0.70-0.90	0.000	0.81	0.71-0.93	0.003
Sex of last baby	Female	1.01	0.89-1.14	0.858	1.04	0.91-1.18	0.573	1.06	0.93-1.21	0.386	1.10	0.96-1.26	0.160
	Male	1 (REF)			1 (REF)			1 (REF)			1 (REF)		
Was baby weighed at birth	Yes	2.01	1.70-2.37	0.000	1.80	1.52-2.13	0.000	2.12	1.78-2.52	0.000	1.89	1.59-2.25	0.000
	No	1 (REF)			1 (REF)			1 (REF)			1 (REF)		
Start time of breastefeeding	Within an hour	1 (REF)			1 (REF)			1 (REF)			1 (REF)		
	>1 hours	0.92	0.79-1.08	0.308	0.71	0.60-0.85	0.000	1.01	0.86-1.19	0.874	0.75	0.63-0.90	0.002
Previous baby death	No child death							1 (REF)			1 (REF)		
	Child death within 24 hours							0.99	0.78-1.27	0.962	1.05	0.80-1.38	0.740
	Child death over 24 hours							0.85	0.73-0.99	0.035	0.90	0.76-1.07	0.228
Mode of delivery for last birth	Caesarean birth	2.63	2.05=3.39	0.000	2.88	2.19-3.78	0.000	3.54	2.60-4.82	0.000	3.83	2.78-5.27	0.000
	Vaginal birth	1 (REF)			1 (REF)			1 (REF)			1 (REF)		
